# The therapeutic efficacy and safety of traditional Chinese medicine injection combined with low-molecular-weight heparin on the acute lower extremity deep venous thrombosis: A protocol for systematic review and network meta-analysis

**DOI:** 10.1097/MD.0000000000028039

**Published:** 2021-12-17

**Authors:** Mulan Zeng, Qian Liao, Yinjie Cui, Hongbo Tang, Maowen Yu

**Affiliations:** a Department Second Ward of General Surgery, Jiangxi Pingxiang People's Hospital, Pingxiang, Jiangxi Province, China; b Department of Nursing, Qingdao Central Hospital, Qingdao, Shandong Province, China; c Department of Clinical Lab, Jintang Hospital, West China Hospital, Sichuan University (Jintang First People's Hospital), Chengdu, Sichuan Province, China.

**Keywords:** acute lower extremity deep venous thrombosis, low molecular weight heparin, network meta-analysis, protocol, traditional Chinese medicine injection combine

## Abstract

**Background:**

Lower extremity deep venous thrombosis (LEDVT) of lower extremities is one of the common clinical diseases. Lack of a timely treatment in the acute stage easily causes pulmonary embolism, thus seriously threatening patients’ life. Low-molecular-weight heparin (LMWH), as a new generation of heparin-based anticoagulant and antithrombotic drug, is now commonly used in the clinical treatment of acute lower extremity deep venous thrombosis (ALEDVT). Relevant randomized controlled trials (RCTs) have reported the therapeutic efficacy and safety of Traditional Chinese medicine injection (TCMJ) combined with LMWH on ALDVT, although their conclusions remain controversial. In addition, the efficacy of various TCMJs has rarely been analyzed and compared. This study aims to evaluate the therapeutic efficacy and safety of TCMJ combined with LMWH on ALEDVT through a network meta-analysis.

**Methods:**

RCTs reporting TCMJ combined with LMWH and LMWH along for the treatment of ALEDVT published before November 2021 will be searched in online databases, including the Cochrane Library, Embase, PubMed, Web of Science, Wanfang, Chongqing VIP Chinese Science and Technology Periodical Database, Chinese Biomedical Literature Database, and Chinese National Knowledge Infrastructure. Two investigators will be independently responsible for literature screening, data extraction, and quality evaluation according to Cochrane's risk of bias assessment tool. R software will be used to perform a network Meta-analysis.

**Results:**

The results of this meta-analysis will be submitted to a peer-reviewed journal for publication.

**Conclusion:**

This study will provide high-quality, evidence-based medical evidence for comparing the therapeutic efficacy and safety of TCMJ combined with LMWH and LMWH alone on ALEDVT.

## 1 Introduction

Lower extremity deep venous thrombosis (LEDVT) is a common disease in vascular surgery, which is commonly seen in post-trauma, post-surgical, and long-term bedridden patients caused by abnormal clotting of blood in the veins of the lower extremities, leading to blood reflux obstruction.^[1–3]^ It is reported that the incidence of LEDVT in hospitalized patients is as high as 12% to 25%.^[4,5]^ Lack of an effective treatment of lower extremity deep venous thrombosis (ALEDVT) in time easily leads to post-thrombotic syndrome and pulmonary artery embolism, and even death.^[6]^ Misdiagnosis or non-treatment causes pulmonary embolism in 90% of patients due to thrombus shedding.^[7]^ Once pulmonary embolism occurs, approximately 10% of patients die within 1 hour,^[8]^ posing a great physical and life-threatening risk.^[9]^

Effective prevention and treatment of ALEDVT have been well concerned in the field of vascular surgery. In recent years, modern research in traditional Chinese medicine has found their efficacy on preventing and treating ALEDVT.^[10]^ Low-molecular-weight heparin (LMWH), as a new generation of heparin-based anticoagulant and antithrombotic drug, has commonly used in clinical treatment of ALEDVT.^[11,12]^ Numerous evidences have shown that herbal injection (TCMJ) combined with LMWH can achieve enhanced efficacy and improve the survival quality of patients with ALEDVT,^[13–17]^ which can be attributed to the improvement of hemodynamics, microcirculation and endothelial cell function, inhibition of platelet aggregation, and scavenge of oxygen free radicals.^[14,18]^ Currently, TCMJ has been widely used in the prevention and treatment of blood stasis evidence and relevant diseases, including ALEDVT.

Network meta-analysis (NMA) is an extension of the traditional meta-analysis, which is able to simultaneously compare the effectiveness of multiple interventions and thus provide better results of systematic evaluations.^[19]^ Due to the wide variety of TCMJs and their different therapeutic focuses, comparison of the individualized efficacy has been rarely reported. Therefore, it is difficult to determine which TCMJ presents the optimal efficacy and safety on ALEDVT. In this study, we will perform NMA to analyze RCTs reporting TCMJ combined with LMWH for the treatment of ALEDVT.

## 2 Methods

### 2.1 Study registration

The protocol of this review will be registered in OSF Registries (OSF registration number: DOI 10.17605/OSF.IO/T65SK), which follows the statement guidelines of preferred reporting items for systematic reviews and meta-analyses protocol.^[20]^

### 2.2 Inclusion criteria for study selection

#### 2.2.1 Types of studies

RCTs reporting TCMJ combined with LMWH versus LMWH alone for the treatment of ALEDVT published in English and Chinese language.

#### 2.2.2 Types of participants

(1) Diagnosis of ALEDVT by vascular ultrasound or angiography (disease onset ≤ 2 weeks);
(2) Older than 18 years;
(3) Patients with contraindications of anticoagulant thrombolytic therapy will be excluded;
(4) No allergy to TCMJ.

#### 2.2.3 Types of interventions and comparators

LMWH alone given to the control group, and TCMJ combined with LMWH given to the experimental group. Drug dose, administration, and duration of treatment will be unlimited.

#### 2.2.4 Outcome measurements

(1) Primary outcomes: total clinical response rate;
(2) Secondary outcomes: fibrinogen (FIB), prothrombin time (PT), and activated partial thromboplastin time (APTT);
(3) Incidence of adverse events.

#### 2.2.5 Exclusion criteria

(1) Duplicate publications;
(2) Literatures with unavailable data;
(3) Full-text literature is not available.

### 2.3 Searching strategy

RCTs reporting TCMJ combined with LMWH and LMWH along for the treatment of ALEDVT published before November 2021 will be searched in online databases, including the Cochrane Library, Embase, PubMed, Web of Science, Wanfang, Chongqing VIP Chinese Science and Technology Periodical Database, Chinese Biomedical Literature Database, and Chinese National Knowledge Infrastructure. Searching strategy will be adjusted in each online database. In addition, references from the included literature and relevant meta-analyses will be manually searched to avoid missing literatures. Searching strategy in the PubMed is summarized in Table 1.

**Table 1:** Search strategy in PubMed database.

Number	Search terms
#1	Venous Thrombosis[MeSH]
#2	Deep Vein Thrombosis[Title/Abstract]
#3	Phlebothrombosis[Title/Abstract]
#4	Thrombosis, Deep Vein[Title/Abstract]
#5	Thrombosis, Venous[Title/Abstract]
#6	Deep Venous Thrombosis[Title/Abstract]
#7	Deep-Vein Thrombosis[Title/Abstract]
#8	Deep-Venous Thrombosis[Title/Abstract]
#9	Deep Vein Thromboses[Title/Abstract]
#10	Deep Venous Thromboses[Title/Abstract]
#11	Deep-Vein Thromboses[Title/Abstract]
#12	Deep-Venous Thromboses[Title/Abstract]
#13	Phlebothromboses[Title/Abstract]
#14	Thromboses, Deep Vein[Title/Abstract]
#15	Thromboses, Deep Venous[Title/Abstract]
#16	Thromboses, Deep-Vein[Title/Abstract]
#17	Thromboses, Deep-Venous[Title/Abstract]
#18	Thromboses, Venous[Title/Abstract]
#19	Thrombosis, Deep Venous[Title/Abstract]
#20	Thrombosis, Deep-Vein[Title/Abstract]
#21	Thrombosis, Deep-Venous[Title/Abstract]
#22	Vein Thromboses, Deep[Title/Abstract]
#23	Vein Thrombosis, Deep[Title/Abstract]
#24	Venous Thromboses[Title/Abstract]
#25	Venous Thromboses, Deep[Title/Abstract]
#26	Venous Thrombosis, Deep[Title/Abstract]
#27	OR/1-26
#28	Chinese herbal injections[Title/Abstract]
#29	Traditional chinese medicine injections[Title/Abstract]
#30	Traditional chinese medicine[Title/Abstract]
#31	OR/28-30
#32	Randomized Controlled Trial[MeSH]
#33	Controlled trial[Title/Abstract]
#34	Random∗[Title/Abstract]
#35	Controlled Clinical Trial[Title/Abstract]
#36	Clinical Trial[Title/Abstract]
#37	OR/32-36
#38	#27 AND #31 AND #37

### 2.4 Data collection and analysis

**Figure 1:**
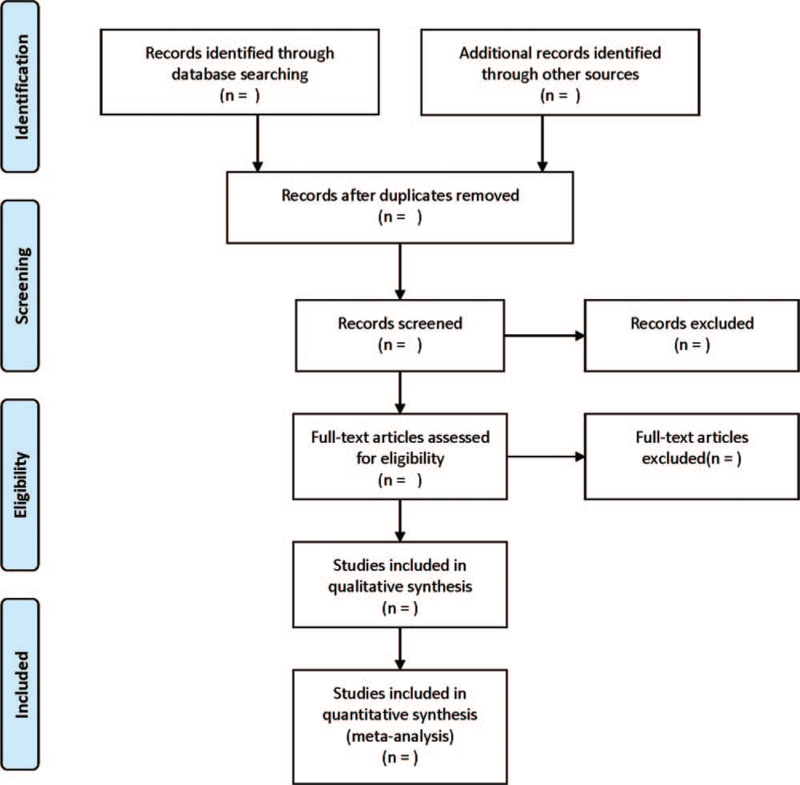
Flow diagram of study selection process.

Two researchers will be independently responsible for literature searching based on the inclusion and exclusion criteria. Any disagreement will be solved by discussing with the third researcher. Searching data after reading the title and abstract will firstly be imported into Endnote X8 software for checking. Then, the full-text of initially searched literatures will be thoroughly reviewed. Searching strategy was displayed in the PRISMA flow chart (Fig. 1).

#### 2.4.1 Data extraction and management

Data extraction will be carried out independently by 2 researchers. Any disagreement will be solved by discussing with the third researcher. The following data will be extracted: first author, year of publication, number of patient cases, gender, age, intervention, duration of treatment, and outcome indicators, etc.

#### 2.4.2 Assessment of risk bias

The quality of included studies will be strictly evaluated in accordance with the Cochrane Risk of Bias Assessment Tool^[21]^ in the following 7 aspects: appropriateness of the method of random sequence generation; concealment of the allocation scheme; blinding of study subjects, implementers; blinding of those assessing the study results; completeness of outcome data; selective reporting of study results; and presence of other sources of bias. Risk bias will be categorized into 3 levels: low risk, unclear, and high risk.

#### 2.4.3 Measures of therapeutic efficacy

The standardized mean difference and its corresponding 95% confidence intervals (95% CIs) of continuous variables will be calculated. The relative risk ratio and its corresponding 95% CIs of binary variables will be calculated as well.

#### 2.4.4 Management of missing data

The reason for missing data during data screening and extraction will be identified. Insufficient, missing, or vague data will be requested by contacting the first author; Otherwise, these studies will be excluded.

### 2.5 Data synthesis and statistical methods

NMA will be performed using R software (version 4.1.2) via calling the gemtc package in conjunction with JAGS software, based on a Bayesian framework using the Markov Chain Monte Carlo (MCMC) method. Simulation analysis will be performed using four Markov chains with an initial value of 2.5, a refinement iteration step of 10, 20,000 pre-simulation iterations, and 100,000 iterations. Deviance information criterion (DIC) values of the random-effects model and fixed-effects model will be compared. If the difference in DIC between the 2 models is ≤5, the fixed-effects model will be used; Otherwise, the random-effects model with a smaller DIC value will be used. In the present study, LMWH monotherapy will be directly compared with the combination treatment of TCMJ and LMWH, rather than a direct comparison of two combined treatments. As a result, there will be no closed loop, and consistency test and model will not be applied. Network evidence relationships, ranked probability bar graphs, ranked probability line graphs, and cumulative ranked probability graphs will be plotted for each outcome indicator. The surface under the cumulative ranking curve (SUCRA) will be used to visualize cumulative ranking probability, and the superiority of the interventions will be ranked according to the magnitude of the SUCRA value.

### 2.6 Assessment of reporting biases

Comparison-adjusted funnel plots will be depicted to identify evidence for small-sample effects on intervention networks.^[22]^

### 2.7 Subgroup analysis

Subgroup analysis will be performed according to the age of the patients, the duration of intervention and the dose of LMWH.

### 2.8 Sensitivity analysis

Sensitivity analysis will be performed to compare the effect size of the combined random-effects model with that of the fixed-effects model to test the stability of NMA results.

### 2.9 Ethics and dissemination

The NMA protocol was approved by the local institutional review board and ethics committee. Ethical approval or informed consent is not needed because private information of patients is not involved in.

## 3 Discussion

ALEDVT can occur in all clinical departments and is prone to complicate pulmonary embolism and even sudden death, and therefore, it is a clinical emergency, and critical and severe condition.^[23–25]^ It is urgent to develop effective prevention and treatment of ALEDVT.

With the development of modern medicine, and various anticoagulation, thrombolytic, and surgical treatments frequently used in clinical practice, LMWH has become a common drug for the treatment of ALEDVT because of its strong anticoagulant and antithrombotic effects, low bleeding risk, and low incidence of thrombocytopenia.^[26]^ ALEDVT belongs to pulse paralysis in Chinese medicine, its pathogenesis of which is linked with the stagnation of Qi and blood, leading to redness, swelling, and pain.^[27]^ The combined treatment of TCMJ and LMWH of ALEDVT has achieved therapeutic efficacy.

To date, most studies have only reported the efficacy of a single TCMJ combined with LMWH in the treatment of ALEDVT, and comparisons between different TCMJs are lacked. Therefore, it is difficult to determine which TCMJ combined with LMWH presents the best efficacy and safety on ALEDVT. This study aims to investigate the efficacy and safety of different TCMJ combined with LMWH on ALEDVT by NMA, and we will rank them according to the superiority of the index effect, thus providing an evidence-based basis for making the best clinical decision.

## Author contributions

**Conceptualization:** Maowen Yu, Mulan Zeng.

**Data curation:** Qian Liao.

**Formal analysis:** Qian Liao, Yinjie Cui.

**Funding acquisition:** Maowen Yu.

**Investigation:** Qian Liao.

**Methodology:** Qian Liao, Yinjie Cui, Hongbo Tang.

**Project administration:** Maowen Yu.

**Resources:** Yinjie Cui.

**Software:** Yinjie Cui, Hongbo Tang.

**Supervision:** Maowen Yu.

**Validation:** Hongbo Tang, Qian Liao.

**Visualization and software:** Qian Liao.

**Visualization:** Hongbo Tang.

**Writing – original draft:** Mulan Zeng and Maowen Yu.

**Writing – review & editing:** Mulan Zeng and Maowen Yu.
